# Development and validation of an LC-MS/MS method for ruxolitinib quantification: advancing personalized therapy in hematologic malignancies

**DOI:** 10.3389/jpps.2024.12905

**Published:** 2024-06-28

**Authors:** Na Li, Huiying Zhang, Haochen Bai, Kaizhi Lu

**Affiliations:** ^1^ Mass Spectrometry Research Institute, Beijing Gobroad Hospital, Beijing, China; ^2^ Mass Spectrometry Research Institute, Beijing Gobroad Healthcare Group, Beijing, China; ^3^ Shanghai Liquan Hospital, Shanghai, China

**Keywords:** ruxolitinib, LC-MS/MS, hematologic malignancies, therapeutic drug monitoring, precision medicine

## Abstract

**Background:**

Hematologic malignancies such as leukemia and lymphoma present treatment challenges due to their genetic and molecular heterogeneity. Ruxolitinib, a Janus kinase (JAK) inhibitor, has demonstrated efficacy in managing these cancers. However, optimal therapeutic outcomes are contingent upon maintaining drug levels within a therapeutic window, highlighting the necessity for precise drug monitoring.

**Methods:**

We developed a sensitive liquid chromatography-tandem mass spectrometry (LC-MS/MS) method to quantify ruxolitinib in human plasma, improving upon traditional methods in specificity, sensitivity, and efficiency. The process involved the use of advanced chromatographic techniques and robust mass spectrometric conditions to ensure high accuracy and minimal matrix effects. The study was conducted using samples from 20 patients undergoing treatment, with calibration standards ranging from 10 to 2000 ng/mL.

**Results:**

The method displayed linearity (*R* ^2^ > 0.99) across the studied range and proved highly selective with no significant interference observed. The method’s precision and accuracy met FDA guidelines, with recovery rates consistently exceeding 85%. Clinical application demonstrated significant variability in ruxolitinib plasma levels among patients, reinforcing the need for individualized dosing schedules.

**Conclusion:**

The validated LC-MS/MS method offers a reliable and efficient tool for the therapeutic drug monitoring of ruxolitinib, facilitating personalized treatment approaches in hematologic malignancies. This approach promises to enhance patient outcomes by optimizing dosing to reduce toxicity and improve efficacy.

## Introduction

Leukemia and lymphoma, the most prevalent forms of blood cancer, are characterized by the rapid proliferation of abnormal white blood cells and lymphocytes, respectively, posing significant challenges for treatment due to their heterogeneity and the potential for resistance to standard therapies [1, 2]. This complexity is compounded by the diseases’ varied incidence across different populations and their capacity to evade long-term control, even with advanced treatment modalities [3–5]. The refractory nature of certain types of leukemia and lymphoma, particularly those that are aggressive or have developed resistance to conventional treatments, underscores the urgent need for innovative therapeutic approaches tailored to the unique genetic and molecular profiles of individual patients [6, 7].

Ruxolitinib, a targeted inhibitor of the Janus kinase (JAK) pathway, has shown promise in the treatment of various hematological malignancies by disrupting the JAK-STAT signaling mechanism, which is often aberrantly activated in these diseases [8, 9]. Its application in conditions such as myelofibrosis and polycythemia vera has demonstrated not only symptomatic relief but also a potential to modify the disease trajectory, offering hope for its utility in leukemia and lymphoma, especially in cases where traditional treatments fall short [10–13]. The specificity of ruxolitinib’s action presents an opportunity to minimize the side effects commonly associated with broader-spectrum chemotherapies, aligning with the goals of precision medicine to provide more effective and less toxic treatment options [14].

The role of therapeutic drug monitoring (TDM) is increasingly recognized as crucial in optimizing treatment outcomes [15, 16], particularly for drugs like ruxolitinib, which have narrow therapeutic ranges. TDM involves the careful measurement of drug levels in the bloodstream to ensure that they remain within a therapeutic range that maximizes efficacy while minimizing toxicity [17, 18]. This approach is especially pertinent in the management of hematologic malignancies, where patient-specific factors such as genetic variations, drug interactions, and coexisting conditions can significantly impact drug metabolism and response [19].

Despite the critical role of TDM, existing methods such as traditional liquid chromatography and conventional mass spectrometry often fall short in meeting the rigorous demands of modern pharmacokinetics in hematologic malignancies [20]. These methods have faced challenges in sensitivity and specificity, often unable to detect lower concentrations of ruxolitinib effectively or differentiate it accurately from similar compounds. Furthermore, these methods can be time-consuming and inflexible, limiting their utility in high-throughput and diverse clinical settings.

To address these limitations, we have developed an LC-MS/MS method that demonstrates superior sensitivity and specificity, capable of detecting lower concentrations of ruxolitinib and reducing the likelihood of interference. Additionally, our method significantly reduces sample throughput time, enhancing efficiency in high-throughput settings. It also shows greater robustness against matrix effects and provides flexibility in adapting to various sample types and conditions—capabilities that are crucial for effectively managing the variability in patient responses and the complex nature of hematologic malignancies.

By tailoring therapy to the individual patient’s pharmacokinetic profile and specific disease characteristics, healthcare providers can enhance the precision and efficacy of treatment, potentially transforming the management of these challenging diseases. This approach not only embodies the principles of precision medicine but also offers a pathway to better outcomes for patients with leukemia and lymphoma, who have historically faced significant obstacles in achieving favorable prognoses. The integration of targeted therapies like ruxolitinib, supported by strategies such as TDM, holds the potential to significantly improve the therapeutic landscape for both leukemia and lymphoma, paving the way for more personalized, effective, and safer treatment modalities.

## Materials and methods

### Reagents

Blank human plasma was provided by the blood bank at the Beijing Gobroad Boren Hospital. Ruxolitinib reference standard (batch number: 2-ATO-31-1-GJZ-63-1, purity ≥98%) and deuterated ruxolitinib (ruxolitinib-d9) reference standard (batch number: 10-MMH-128-2, purity ≥97%) were both acquired from TRC Canada. Methanol (HPLC grade) was obtained from Fisher Scientific, United States. Formic acid (HPLC grade) was sourced from Thermo Fisher Scientific, Shanghai, China. Water was Watsons distilled water, provided by Guangzhou Watsons Distilled Water Co., Ltd.

### Instrumentation and chromatographic/mass spectrometric conditions

The analysis of ruxolitinib in plasma samples was performed using a Thermo Ultimate 3000 UHPLC system paired with a Thermo Ultimate 3000 UHPLC mass spectrometer (Thermo Fisher Scientific, Waltham, MA, United States), renowned for its high sensitivity and throughput, ideal for the detection of low-concentration analytes such as ruxolitinib.

Chromatographic separation was achieved on a Thermo Hypersil GOLD C18 column (50 mm × 2.1 mm, 3.0 µm), selected for its robust resolution and chemical stability. The column’s performance was enhanced by a guard column, ensuring consistent chromatographic results over time. The LC-MS method utilized a mobile phase comprising 0.1% formic acid in water (A) and 0.1% formic acid in methanol (B), using a gradient elution strategy designed to enhance the retention and differentiation of ruxolitinib and its internal standard Ruxolitinib-13C9 within the biological matrix. The flow rate was meticulously set at 0.4 mL/min, with the column oven temperature regulated at 40°C to maintain optimal separation conditions. The gradient elution begins with an equilibration phase, maintaining 15% of solvent B for the first minute. The percentage of solvent B then sharply rises to 85% over the next minute and is held constant until 2.2 min. Following this, the gradient returns to 15% solvent B at 2.3 min, re-establishing the initial conditions for re-equilibration until the conclusion of the run at 3 min.

Mass spectrometric detection was carried out in positive electrospray ionization (ESI+) mode, employing selected reaction monitoring (SRM) to target specific m/z transitions for ruxolitinib and the internal standard, thereby ensuring selectivity and minimizing matrix interferences [21]. Detailed mass spectrometry conditions for rucotinib are shown in Table 1. Instrumental parameters, such as declustering potential and collision energy, were finely tuned to achieve the highest sensitivity and specificity for ruxolitinib detection. The temperature of the autosampler was maintained at 4°C to ensure the stability of samples during analysis.

**TABLE 1:** Mass spectrometric conditions for ruxolitinib quantitation.

Compound	Q1	Q3	Dwell time (s)	Collision energy (V)	RF lens (V)
Ruxolitinib	307.1	186.1	0.3	54	50
Ruxolitinib-13C9	316.1	186.1	0.3	54	50

Data acquisition, management, and analysis were conducted using the integrated Thermo Scientific software, providing a robust platform for peak integration, quantitative analysis, and ensuring data integrity in compliance with established analytical standards.

### Preparation of calibration standards and QC samples

Ruxolitinib calibration standards were prepared by serial dilution of stock solutions in blank human plasma, covering a concentration range of 10–2000 ng/mL. Quality Control (QC) samples were formulated at low, medium, and high concentrations within this calibration range to ensure comprehensive monitoring of the assay’s accuracy and precision throughout the analytical range.

### Sample preparation

Blood samples were collected 30 min prior to the fourth drug administration to measure ruxolitinib concentrations. A 100 µL aliquot of each plasma sample was fortified with 300 µL of methanol containing the internal standard to precipitate proteins. The mixture was vortexed vigorously and centrifuged at 14,000 g for 10 min to obtain a clear supernatant. The supernatant was then transferred to a new sample vial and a 1 µL aliquot was injected into the UHPLC-MS/MS system for ruxolitinib quantification.

### Clinical platelet monitoring

Simultaneously with the ruxolitinib concentration measurements, blood samples for platelet monitoring were drawn into EDTA tubes. Platelet counts were analyzed using automated hematology analyzers. This critical data informed ruxolitinib dosage adjustments to mitigate the risks of thrombocytopenia. Quality control checks were performed to ensure the accuracy of the platelet measurements, aligning with the study’s objective to monitor and adjust treatment based on comprehensive clinical parameters. All experimental procedures were conducted following an ethical approval granted by the Ethics Committee of Beijing Gobroad Hospital.

### Method validation

The validation of the analytical method adhered to FDA bioanalytical method validation guidelines, assessing specificity, linearity, accuracy, precision, recovery, matrix effect, and stability [22, 23]. The method demonstrated excellent linearity with a coefficient of determination (*R* ^2^) consistently exceeding 0.99. Accuracy and precision were evaluated across multiple analytical runs, with performance within the acceptable range of ±15% deviation from the nominal values, and within ±20% at the lower limit of quantification (LLOQ).

The acceptance criteria for the validation of our LC-MS/MS method to quantify ruxolitinib are comprehensive and ensure the method’s robustness and reliability. Specificity is confirmed by analyzing chromatograms from six different sources of blank plasma spiked with ruxolitinib and its internal standard, ensuring no interference from other substances. The accuracy of the assay is maintained within 85–115% of the nominal concentration across all levels, except at the (LLOQ, where it is within 80–120%. Precision is kept under 15% CV% for standard concentrations and under 20% at LLOQ. The calibration curve demonstrates linearity with *R* ^2^ ≥ 0.99, and standard concentrations deviate no more than 15% from nominal values, except at LLOQ (20%). Recovery is consistent, precise, and reproducible across the QC sample range. Matrix effects are minimized, assessed by comparing the response of matrix-prepared samples to those prepared in solvent. Stability tests confirm that ruxolitinib remains stable under various conditions including bench-top, freeze/thaw, and long-term storage, with stability established at both initial and end concentrations. Additionally, robustness is ensured as small deliberate changes in method parameters do not significantly affect the assay outcome, supporting the method’s applicability under varied conditions and its compliance with regulatory standards.

### Statistical analysis

Statistical analyses were conducted using SPSS version 22.0 (IBM Corp., Armonk, NY, United States). Pharmacokinetic parameters for ruxolitinib were derived using non-compartmental analysis techniques. The relationship between ruxolitinib plasma concentrations and platelet counts was examined using Pearson’s correlation coefficient. Variability in pharmacokinetic parameters across different dosing regimens or patient subgroups was assessed using appropriate statistical tests, such as ANOVA or Kruskal-Wallis tests, with a significance threshold set at *p* < 0.05.

## Results

### Selectivity

**FIGURE 1:**
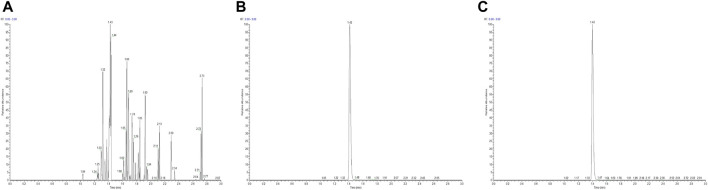
High-performance liquid chromatography chromatogram of ruxolitinib [**(A)** Blank plasma, **(B)** Blank plasma + ruxolitinib, **(C)** Test plasma].

The selectivity of the LC-MS/MS method for the quantification of ruxolitinib in human plasma was evaluated using six distinct blank plasma samples sourced from different donors. These samples were prepared both as blank matrices and as matrices spiked with ruxolitinib at the lower limit of quantification (LLOQ), followed by the prescribed sample preparation procedures. Subsequent analysis revealed that ruxolitinib eluted at a consistent retention time of approximately 1.42 min across all samples. Investigation of potential interfering peaks at the retention time of ruxolitinib indicated that the response of any such peaks in the blank plasma samples was below 20% of the response observed for ruxolitinib in the LLOQ samples. Similarly, at the retention time of the internal standard, the interference peak responses were found to be less than 5% of the internal standard response in the zero-concentration (blank) samples (Figure 1). These findings suggest negligible interference from the plasma matrix in the quantification of ruxolitinib and the internal standard, confirming the method’s high selectivity.

### Internal standard recovery

Internal standard recovery was evaluated in normal plasma conditions. The recovery rates were highly consistent, demonstrating the method’s effectiveness and precision. This ensures the method’s reliability for accurate quantification of ruxolitinib in clinical settings. Detailed information is shown in Table 2.

**TABLE 2:** Internal standard recovery (n = 6).

Concentration type	Peak area (Pre-Extraction)	Peak area (post-extraction)	Mean recovery (%)	SD	CV (%)	Overall recovery %	Standard deviation	Overall variation %CV
Low QC	519,482	540,590	96.1	2.68	2.79	97.16	1.5	1.54
High QC	564,335	556,235	98.2	1.72	1.75			

QC: quality control; SD: standard deviation; CV: coefficient of variation.

### Dilution integrity

The integrity of the dilution effect was evaluated by preparing quality control (QC) samples at two dilution factors, 2-fold (demonstrate a concentration of 2400 ng/mL) and 10-fold (demonstrate a concentration of 4000 ng/mL), to test the method’s accuracy and precision under these conditions. This assessment is crucial for cases where sample concentrations exceed the upper limit of quantification and require dilution for accurate analysis. The results demonstrated that the method maintained its accuracy and precision across both dilution levels, indicating the robustness of the method in handling samples with high concentrations of ruxolitinib. Specific results are shown in Table 3.

**TABLE 3:** Summary statistics for dilution integrity transposed (n = 6).

Metric	Mean (ng/mL)	SD	CV%	Accuracy (%)	DEV%
High Concentration (10x dilution)	408.51	8.2	2.01	102.13	3.84
Low Concentration (2x dilution)	1256.18	11.14	0.89	1046.82	−0.33

SD: standard deviation; CV: coefficient of variation; DEV:deviation from expected.

### Carryover validation

The evaluation of carryover effects was performed using a sequence of analyses that included blank plasma samples, low concentration samples, and high concentration samples set at the upper limit of quantification (ULOQ). The sequence began with the analysis of a high concentration sample to assess the system’s ability to return to a baseline state without residual effects influencing subsequent blank samples. The findings, as summarized in Table 4, indicated that the first and second blank samples following the high concentration ruxolitinib sample exhibited carryover rates of 1.20% and 0.60%, respectively. For the internal standard, the carryover in the first and second blank samples post high concentration sample analysis was determined to be 0.10%. These results demonstrate that the carryover in this LC-MS/MS method for ruxolitinib quantification is negligible, thereby not impacting the accuracy of quantitative analysis.

**TABLE 4:** Evaluation results of ruxolitinib residual experiment (n = 6).

Sample type	Analyte			Internal standard		
	Peak area	LOQ peak area	Residue	Peak area	LOQ peak area	Residue
ULOQ	1,409,694	15,733	-	100,371	112,441	-
Blank Plasma-1	195		1.20	172		0.10
Blank Plasma-2	95		0.60	208		0.10

ULOQ: upper limit of quantitation; LOQ: limit of quantitation.

### Matrix effect

The matrix effect for ruxolitinib quantification in human plasma was comprehensively evaluated by analyzing quality control (QC) samples at low and high concentration levels, prepared in both blank plasma from six different sources and in water. The evaluation utilized internal standard-normalized matrix factors to assess the extent of ion suppression or enhancement resulting from the plasma matrix. For normal, hemolyzed, and lipemic plasma, the mean matrix factors ranged from 0.80 to 1.00, indicating minimal matrix effects across different biological matrices. The results are shown in Table 5.

**TABLE 5:** Internal standard normalized matrix factor of ruxolitinib in different plasma conditions (n = 6).

Condition	LQC			HQC		
	Mean	SD	CV%	Mean	SD	CV%
Normal	0.85	0.01	1.02	0.90	0.05	5.45
Hemolyzed	0.85	0.00	0.28	0.91	0.01	1.07
Lipemic	0.85	0.01	0.76	0.80	0.01	0.66

LQC: low quality control; HQC: high quality control; SD: standard deviation; CV: coefficient of variation.

### Extraction recovery

The extraction recovery of ruxolitinib from human plasma was determined by analyzing QC samples prepared at three concentration levels (low, medium, and high) in both plasma and post-extraction plasma supernatant. This approach allowed for the assessment of the efficiency with which ruxolitinib and its internal standard were recovered from the plasma matrix during the sample preparation process. The results indicated that the extraction recovery rates for ruxolitinib across the tested concentration levels ranged from 88.47% to 93.24%. These values reflect the consistency and efficiency of the extraction process employed in this method, ensuring that a high proportion of the analyte is recovered from the plasma matrix for subsequent analysis. The high extraction recovery rates demonstrate the effectiveness of the sample preparation procedure in isolating ruxolitinib from complex biological matrices. The results are shown in Table 6.

**TABLE 6:** Summary of ruxolitinib extraction recovery rates (n = 6).

Name	QCL	QCM	QCH
	Extraction recovery rate (%)	Extraction recovery rate (%)	Extraction recovery rate (%)
Ruxolitinib	88.47 ± 1.50	88.95 ± 1.25	93.24 ± 0.85

QCL: quality control low; QCM: quality control medium; QCH: quality control high.

### Precision and accuracy

The precision and accuracy of the LC-MS/MS method for quantifying ruxolitinib in human plasma were evaluated using quality control (QC) samples at four concentration levels: the lower limit of quantification (LLOQ), low, medium, and high (QCL, QCM, and QCH, respectively). These samples were prepared in blank plasma and analyzed over 3 days, with three different batches per day, to assess both intra-day and inter-day variability. The accuracy of the method, expressed as the relative error (RE), was found to be within the range of 91.04%–114.21% across all concentration levels. This range indicates that the quantification of ruxolitinib is generally reliable and conforms closely to the true values. The intra-day precision, as indicated by the relative standard deviation (RSD), did not exceed 6.46%, while the inter-day precision was within 7.50%. These RSD values underscore the method’s consistency in quantifying ruxolitinib on both a daily basis and across different days. Precision and accuracy within the acceptable ranges confirm the method’s suitability for the reliable and consistent quantification of ruxolitinib in human plasma. The results are presented in Table 7.

**TABLE 7:** Summary of precision and accuracy data for ruxolitinib (n = 6).

Name		First batch		Second batch		Third batch		Inter-batch	
	Level	CV/%	RE/%	CV/%	RE/%	CV/%	RE/%	CV/%	RE/%
Ruxolitinib	QCL	4.27	104.75	3.91	97.11	6.46	91.04	7.50	97.64
	QCM	1.15	113.39	4.08	99.47	2.86	98.44	7.27	103.77
	QCH	0.85	114.21	5.82	101.14	2.49	100.28	7.07	105.21

QCL: quality control low; QCM: quality control medium; QCH: quality control high; CV: Intra-batch precision; RE: relative error.

### Stability

The stability of ruxolitinib in human plasma was investigated by assessing quality control (QC) samples at three concentration levels (low, medium, and high) after specific storage conditions. The samples were prepared in blank plasma and subjected to pre-treatment processes to evaluate their stability when stored at room temperature for 4 h and in the autosampler for 24 h. The results demonstrated that ruxolitinib-containing plasma samples remained stable when stored at room temperature for up to 4 h, with the accuracy of the quantification ranging between 97.44% and 111.58%. Furthermore, the samples were found to be stable after 24 h of storage in the autosampler, with accuracy levels ranging from 99.92% to 110.64%. These findings indicate that ruxolitinib does not undergo significant degradation under these conditions, ensuring reliable quantification after sample processing and during analytical runs.

The observed stability of ruxolitinib in plasma samples under both storage conditions is crucial for practical analytical workflows, allowing for flexibility in sample processing and analysis without compromising the accuracy of the quantification. This attribute enhances the method’s applicability in clinical settings, where sample storage and handling conditions may vary. Specific results are shown in Table 8.

**TABLE 8:** Summary of analyte stability in ruxolitinib plasma matrix samples (n = 6).

Name	Examination conditions	QCL		QCM		QCH	
		CV/%	RE/%	CV/%	RE/%	CV/%	RE/%
Ruxolitinib	Room Temperature for 4 h	4.29	97.44	1.84	108.11	4.08	111.58
	Autosampler Storage for 24 h	2.31	99.92	5.22	108.34	2.65	110.64

QCL: quality control low; QCM: quality control medium; QCH: quality control high; CV: Intra-batch precision; RE: relative error.

### Standard curve and lower limit of quantification

The construction of the standard curve for ruxolitinib quantification in human plasma was achieved by preparing and pre-treating quality control (QC) samples at six concentration levels (STD01 through STD06). The linear relationship between the concentration of ruxolitinib in plasma and the peak area ratio (analyte to internal standard) was established using weighted (w = 1/x^2^) least squares linear regression. This approach resulted in a standard calibration equation of the form y = a+bx, where y represents the peak area ratio and x denotes the concentration of ruxolitinib in plasma. The standard curve for rucotinib is shown in Table 9.

**TABLE 9:** Standard curve for ruxolitinib (n = 6).

Compound	Equation	*R* ^2^	Quantitation range
Ruxolitinib	y = 0.00598x-0.0011	0.9949	10–2000 ng/mL
	y = 0.00568x-0.0094	0.9931	
	y = 0.00973x+0.0336	0.9919	

*R* ^2^: coefficient of determination.

Over the course of three consecutive days, one standard curve was generated each day using HPLC-MS/MS, leading to the establishment of ruxolitinib’s linear range. The results confirmed that ruxolitinib exhibits a strong linear relationship within the concentration range of 10–2000 ng/mL. The lower limit of quantification for ruxolitinib was determined to be 10 ng/mL, indicating the method’s sensitivity and its capability to accurately quantify low concentrations of ruxolitinib in human plasma.

### Clinical application

The clinical utility of our newly developed LC-MS/MS method for the quantification of ruxolitinib was validated through an analysis of blood samples from 20 hospitalized patients undergoing ruxolitinib treatment across multiple centers. The method’s ability to precisely measure plasma concentrations of ruxolitinib showcased significant variability among patients, which can be attributed to individual differences in drug metabolism, dosage, age, gender, and specific clinical diagnoses.

**FIGURE 2:**
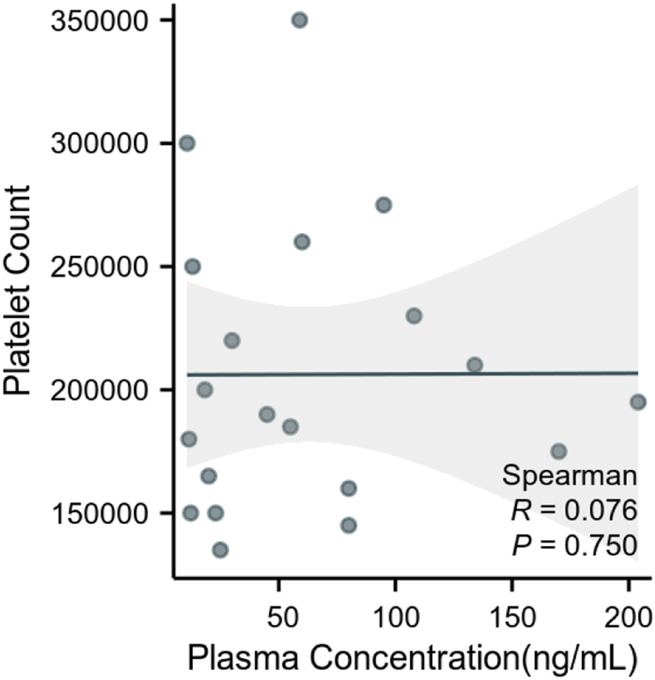
Correlation between plasma ruxolitinib concentration and platelet count in patients with hematologic malignancies.

The clinical application was particularly illustrated by the measured plasma concentrations of ruxolitinib, which varied widely among the patients, from as low as 10.76 ng/mL to as high as 204 ng/mL. This variability underscores the necessity of therapeutic drug monitoring to tailor treatment regimens to individual patient needs. Furthermore, our analysis revealed no significant correlation between ruxolitinib trough concentrations and platelet counts (Figure 2). Despite this, platelet count and drug concentration data were instrumental in guiding dose adjustments. For instance, patients exhibiting sub-therapeutic drug levels underwent dose increases, whereas those on the higher spectrum had their doses maintained or slightly adjusted to optimize therapeutic efficacy and minimize potential toxicity. Patient details are provided in Supplementary Material S1.

## Discussion

The enhancement of the LC-MS/MS methodology for the determination of ruxolitinib levels in human plasma marks a notable achievement in the field of therapeutic drug monitoring, especially pertinent to the management of hematologic malignancies [24, 25]. This refined approach, characterized by its outstanding sensitivity and specificity, meticulously addresses the intricate pharmacokinetic profile of ruxolitinib. Given ruxolitinib’s critical role in treating these cancers and its narrow therapeutic range, the method provides a vital tool for clinicians, enabling them to navigate the complexities of dosing and therapeutic management with greater precision and confidence [26, 27]. Notably, the selectivity of the method minimizes interference from the plasma matrix, a crucial factor considering the potential influence of endogenous substances on drug level measurements [28]. This accuracy is vital for managing the variability in patient responses attributed to genetic, physiological, and disease-specific factors [29]. Additionally, our LC-MS/MS method demonstrates superior sensitivity and specificity, capable of detecting lower concentrations of ruxolitinib and reducing the likelihood of interference. Furthermore, our method significantly reduces sample throughput time, enhancing efficiency in high-throughput settings, and shows greater robustness against matrix effects. It also provides flexibility in adapting to various sample types and conditions—capabilities that are crucial for effectively managing the variability in patient responses and the complex nature of hematologic malignancies. This addition provides readers with a clearer context for the necessity of our methodological developments, demonstrating significant advancements over existing techniques.

These clinical findings significantly enhance our understanding of ruxolitinib pharmacokinetics and the critical role of drug monitoring in personalized medicine. They also support the robustness of our LC-MS/MS method in providing reliable and accurate drug level measurements essential for optimizing treatment regimens in a real-world clinical setting. This approach not only aids in achieving desired therapeutic outcomes but also in reducing the incidence of adverse drug reactions, thereby improving patient safety and treatment efficacy. The clinical implications of accurately quantifying ruxolitinib are profound, especially given the risks associated with high drug concentrations [30]. Higher levels of ruxolitinib are linked to increased adverse effects, necessitating precise measurement to optimize therapeutic efficacy while mitigating toxicity [31, 32]. This approach is pivotal in ensuring personalized and safe treatment regimens, enhancing overall patient outcomes [31].

Our findings, highlighting the method’s robust recovery rates and stability across various storage conditions, reinforce its applicability in diverse clinical settings. The method’s resilience against potential pre-analytical variances is critical in real-world clinical practice, where sample handling and storage conditions may vary. Furthermore, this study opens avenues for future exploration. The method’s adaptability to other JAK inhibitors could be investigated, potentially broadening its clinical utility. Integrating this approach into routine clinical practice might revolutionize the management of hematologic malignancies, aligning treatment strategies more closely with the ideals of precision medicine.

Despite these strengths, our study has certain limitations that must be acknowledged. While we have expanded our cohort to include 40 patients, this sample size, although improved, remains relatively small. This limitation may affect the generalizability of our findings and restricts the scope of the conclusions that can be confidently drawn. Specifically, the modest number of subjects might not fully represent the wider population affected by hematologic malignancies, which could influence the reproducibility and applicability of our results in broader clinical settings. Additionally, our current study focused primarily on trough concentrations of ruxolitinib. Although trough levels are critically linked to therapeutic efficacy and adverse effects, we recognize that a comprehensive pharmacokinetic profile, including a time-course study of plasma drug concentrations at various intervals post-administration, would provide a more robust validation of the pharmacokinetic models. The omission of such a time-course study in the current research could be viewed as a limitation in fully assessing the method’s applicability across different pharmacokinetic scenarios. Acknowledging this, we are committed to incorporating these aspects in future studies to enhance the methodological robustness of our LC-MS approach. As such, while our findings are promising, they should be interpreted with caution, and further studies with larger, more diverse populations are necessary to confirm these results and extend their applicability.

In summary, our newly developed LC-MS/MS method for quantifying ruxolitinib enhances therapeutic drug monitoring in hematologic malignancies with high sensitivity and specificity, crucial for personalized medicine. It allows for precise dosing adjustments, optimizing treatment efficacy and safety. Despite promising results, the small study cohort highlights the need for further research with a larger, more diverse group to ensure broader applicability and to potentially extend this methodology to other JAK inhibitors, thereby advancing personalized treatment strategies in hematologic cancers.

## Data Availability

The original contributions presented in the study are included in the article/Supplementary Material, further inquiries can be directed to the corresponding author.
